# Rethinking Microbial Chemical Ecology: Secondary Metabolites as Concentration-Dependent Signaling Hubs with Implications for Anti-Virulence Intervention

**DOI:** 10.3390/microorganisms14051074

**Published:** 2026-05-09

**Authors:** Jiayuan Cheng, Zhenhua Zhao, Binglu Teng, Wenqing Zhang, Yuanchi Wang

**Affiliations:** Key Laboratory of Integrated Regulation and Resource Development on Shallow Lake of Ministry of Education, College of Environment, Hohai University, Nanjing 210098, China

**Keywords:** quorum sensing, signaling molecules, secondary metabolites, signaling networks

## Abstract

Microorganisms construct complex social communities through the exchange and interaction of chemical substances. Traditional research has typically drawn a strict distinction between quorum-sensing (QS) signaling molecules and cytotoxic secondary metabolites; however, this simplistic classification limits our in-depth understanding of microbial chemical ecology and complex collective behavior. Recent studies have shown that many secondary metabolites exhibit dual functions, acting as signaling molecules that facilitate information exchange at low concentrations. This paper proposes an integrated signaling network framework that views secondary metabolites as key nodes linking microbial collective behavior and environmental adaptation. We explore how this network mechanism overcomes the limitations of linear signaling models, thereby elucidating how microorganisms balance cell growth and metabolite synthesis in dynamic environments. We also introduce emerging spatial omics and synthetic biology tools, which hold great potential for precisely deciphering complex chemical signaling networks at the microscopic scale. Translating these mechanisms into technological applications could enable dynamic, autonomous control of bacterial metabolism in industrial biotechnology, significantly enhancing the yield of target products. Finally, we emphasize the critical importance of reframing chemical ecology as a dynamic signaling network. This shift in ecological and evolutionary perspective not only provides novel intervention pathways based on network decoupling to address the increasingly severe crisis of antibiotic resistance (AMR) but also establishes a theoretical foundation for host microbiome regulation, environmental bioremediation, and industrial multi-strain collaborative engineering.

## 1. Introduction

In natural environments, microorganisms rarely exist as isolated individuals; instead, they form highly structured communities mediated by complex chemical communication. The molecular basis for this social behavior primarily relies on QS mechanisms. Specifically, microorganisms accurately assess the density of surrounding populations by secreting and recognizing specific small molecules; when the concentration of these chemical molecules reaches a critical threshold, the microbial population synchronously alters its gene expression. This communication mechanism enables microorganisms to collectively execute complex behaviors, such as biofilm formation, secretion of virulence factors, and responses to environmental stressors [1,2]. Traditional views of microbiology tend to attribute a single signaling function to QS molecules, while classifying secondary metabolites such as antibiotics and toxins as defensive weapons [3]. Although this dichotomy is straightforward, it overlooks the multiple ecological functions of chemical molecules at different concentrations and under varying environmental conditions, thereby severely limiting our understanding of the complexity of microbial societies.

In recent years, with advances in analytical techniques, a growing body of evidence from ecology and molecular biology has shown that the line between weapons and signals is very blurred. In nature, the local concentrations of secondary metabolites are often far below lethal doses; at these sub-inhibitory concentrations, they effectively function as signaling molecules [4,5]. Not only can they induce changes in the transcriptional state of neighboring cells, but they can also trigger extensive chemical dialogues within multi-species communities. Some researchers have noted that antibiotics secreted by certain bacteria can serve as signals, prompting neighboring species to alter their movement patterns or accelerate escape responses [6,7]. This dual function of secondary metabolites reveals the complex ecological balance between competition and cooperation among microorganisms [8].

Based on these latest insights, this paper proposes an integrated signaling network framework. This framework moves beyond traditional linear thinking, eschewing the view of microbial chemical behavior as merely isolated signal transduction or weaponized attacks. This paper systematically elucidates how secondary metabolites function as critical network nodes that connect and regulate microbial collective behavior. Furthermore, it explores how this dynamic network achieves the decoupling between growth and production, and how industry can leverage this natural network logic to enable dynamic, autonomous control in synthetic biology. Through this framework, we aim to provide a novel perspective for understanding the chemical ecology of microorganisms.

## 2. The QS Functions of Secondary Metabolites

### 2.1. The Signaling Role of Antibiotics

When discussing the dual functions of secondary metabolites, the phenazine compound pyocyanin, secreted by *Pseudomonas aeruginosa*, is the most representative example. For a long time, pyocyanin was regarded solely as a virulence factor and natural antibiotic that induces oxidative stress, disrupts host cells, and inhibits competing microorganisms. However, research by Dietrich et al. has reshaped this understanding. At sub-inhibitory concentrations, pyocyanin acts as a terminal signaling molecule in the QS network, regulating multiple downstream gene clusters including efflux pumps by specifically activating the transcription factor SoxR [9]. This regulation goes beyond simple antioxidant defense and plays a critical role in the late stages of biofilm development. When population density reaches a threshold and nutrients are gradually depleted, pyocyanin functions not only as a biochemical weapon to eliminate competitors but also as a synchronizing signal within the population. It coordinates shifts in cellular metabolic states, promoting the maturation and maintenance of biofilm structures. This seamless transition from exogenous antagonism to endogenous signaling demonstrates how secondary metabolites integrate into the classical Las and Rhl QS regulatory circuit, forming a dual-functional mechanism that confers adaptive advantages.

Although *P. aeruginosa* provides a rich research model for chemical communication, the role of secondary metabolites as concentration-dependent signaling hubs is universally applicable across a wide range of Gram-positive and Gram-negative bacteria. Schoenborn’s research also indicates that various *Bacillus subtilis*-specific metabolites act as intraspecific developmental signals, stimulating biofilm formation and spore formation [10]. Research has shown that surfactin, a lipopeptide secreted by *Bacillus subtilis*, not only possesses antimicrobial activity but also acts as an interspecies signaling molecule to regulate biofilm formation. Across different Bacillus species, surfactin exerts differential regulatory effects through the surfactin-*Spo0A*-*SinI*-*SinR*/*SlrR* signaling pathway. Among these, *spo0A* and *slrR* are essential for biofilm formation in all tested strains, whereas *sinI* and *sinR* exhibit species-specific regulatory patterns [11,12]. In addition to *Bacillus subtilis*, in *Streptococcus mutans*, the secretion of bacteriocins, sensitivity-stimulating peptides that dynamically coordinate genetic transformation and mutation, is regulated to facilitate niche competition [13]. In actinomycetes, γ-butyrolactone (GBL) produced by *Streptomyces* is a highly representative molecule. GBL not only regulates the production of secondary metabolites but also directly controls the formation and morphological differentiation of aerial hyphae in actinomycetes [14,15]. In other Gram-negative bacteria, the diffusible signaling factor (DSF, cis-11-methyl-2-dodecenoic acid) serves as a central signal for *Xanthomonas campestris* to regulate virulence and exopolysaccharide synthesis; more importantly, it mediates cross-species communication, thereby altering the phenotype of coexisting pathogens [16]. He et al. found that in clinical isolates of *Acinetobacter baumannii*, antibiotics such as levofloxacin or meropenem act as signals to induce the overexpression of efflux pumps, stimulating the release of acetylated hyaluronate, which, in turn, enhances QS-mediated biofilm formation and increases AMR [17]. In this context, the responses to antibiotic stress and morphological transformation are tightly coupled within the same signaling network, ensuring the survival and spread of microorganisms under adverse conditions. Additionally, Kalia et al. revealed that plant secondary metabolites exhibit anti-biofilm activity against *Escherichia coli* O157:H7 that is dependent on quorum quenching (QQ) [18].

**Figure 1:**
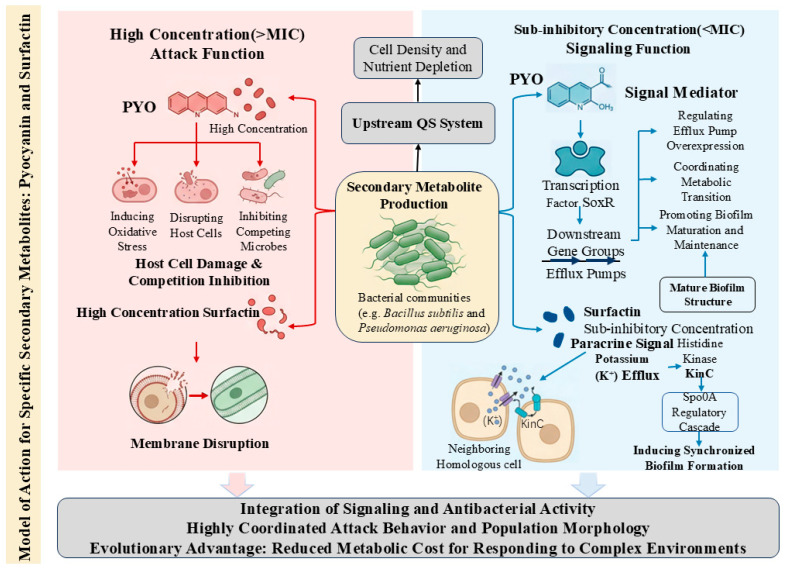
Dual-function mechanism of secondary metabolites. This model illustrates the specialized function of the secondary metabolites Pyocyanin and Surfactin, produced by specific bacteria, and is not a general model for all secondary metabolites. The red arrows represent Pyocyanin, and the blue arrows represent Surfactin.

Integrating signal transduction and antimicrobial activity into a single molecule significantly reduces the metabolic cost for microorganisms to adapt to complex environments from an evolutionary perspective (Figure 1). Within these integrated networks, these molecules link different physiological states of bacteria. They receive instructions from upstream QS systems and subsequently amplify this state within the population or even across communities through positive feedback or paracrine mechanisms, ultimately achieving a high degree of coordination between aggressive behavior and group morphology. This analysis of interactions demonstrates that microbial chemical communication constitutes a dynamic, multifunctional network. Without considering the signaling properties of antibiotics, it is impossible to fully comprehend the sophistication of microbial social behavior.

### 2.2. Antibacterial Activity of Signaling Molecules

As mentioned earlier, secondary metabolites can act as signaling molecules at low concentrations (Section 2.1). In microbial environments, however, this role reversal is bidirectional: classic QS signaling molecules also exhibit potent antibiotic activity or direct toxic effects under specific conditions. This phenomenon, in which signaling molecules become weapons, further blurs the absolute boundaries between signal transduction and chemical defense in traditional microbiology, demonstrating the high economy and multifunctionality of molecular evolution [19,20].

Short-chain N-acylhomoserine lactones (AHLs) secreted by *Chromobacterium violaceum* are believed to serve purely as signaling molecules that regulate the synthesis of violacein, a secondary metabolite with potent antibacterial activity [21]. However, AHL molecules with specific structures spontaneously undergo structural changes in the environment; AHL molecules bearing a 3-oxo substituent can spontaneously rearrange to form tetramic acids [22,23]. Tetramic acids are a key structural component of broad-spectrum natural antibiotics, capable of specifically disrupting cell wall synthesis and membrane potential in Gram-positive bacteria. This non-enzymatic conversion of signaling molecules into potent antibiotics enables AHL-producing strains to achieve the dual ecological goals of communication and antagonism through a single parent molecule without incurring additional biosynthetic energy costs [23].

Short-chain AHLs typically exhibit high water solubility and diffusivity, allowing them to traverse biological membranes freely. Consequently, short-chain AHLs tend to function as signaling molecules, ensuring rapid and widespread signal synchronization even at low population densities [24,25]. In contrast, long-chain AHLs exhibit significant hydrophobicity and transmembrane insertion capabilities; these altered physicochemical properties confer stronger toxicological potency on long-chain AHLs. During infection, long-chain AHLs act as potent immunomodulators and cytotoxins, inducing host cell apoptosis or suppressing immune responses [26,27].

QS signaling molecules and antibiotics are not two distinct categories of substances; from the highly efficient communication mediated by short-chain AHLs to the antibacterial toxicity of long-chain AHLs and their derivatives, this diversity in structure and function demonstrates that microorganisms have evolved a strategy of multifunctionality through prolonged competition [23,24].

### 2.3. Signaling Functions of Other Secondary Metabolites

Building on the previous discussion of the dual functions of classical antibiotics and AHL-type chemotactic signaling molecules, this section will further expand to cover several other major classes of key secondary metabolites in microbial communities: bacteriocins, iron carriers, and pigments. These molecules, which are typically regarded as having single functions, also exhibit indispensable signaling functions in complex microbial environments, collectively forming multidimensional chemical-ecological networks of microorganisms [28].

Bacteriocins are a class of antimicrobial peptides synthesized by bacterial ribosomes; they are regarded as highly specific, targeted weapons used to eliminate competing neighbors [29]. However, at sub-inhibitory concentrations, both nisin from *Lactococcus lactis* and subtilin from *Bacillus subtilis* exhibit QS autoinduction characteristics. Not only do they disrupt the membranes of target cells, but they also act as signaling molecules that bind to histidine kinase receptors on their own cell membranes, activating the expression of their own biosynthetic gene clusters via a two-component system [30,31]. This mechanism ensures that bacteria expend significant energy on weapon synthesis only under high cell density and competitive pressure, thereby conserving metabolic energy.

Iron carriers are high-affinity iron chelators secreted by microorganisms in iron-deficient environments; in addition to their basic role in nutrient uptake, they have been shown to function as important extracellular signaling molecules in numerous pathogenic bacteria [32]. After pyoverdine, secreted by *P. aeruginosa*, chelates iron extracellularly, it binds to the outer membrane receptor FpvA, not only mediating iron uptake but also activating the selective *σ* factor PvdS through a transmembrane signaling cascade [33]. PvdS, in turn, upregulates the expression of various virulence factors, including exotoxin A and endopeptidases [34,35]. This signaling mechanism, which closely links environmental nutrient sensing with virulence expression, positions iron carriers not merely as simple chelators but as critical hubs regulating host-pathogen interactions.

Microbial pigments also possess rich signaling implications. Phenazine pigments are not only respiratory toxins and electron acceptors but also serve as intracellular redox-state signals that can penetrate biological membranes [36]. Research by Dietrich et al. indicates that phenazine pigments can directly regulate transcription factors by altering intracellular redox homeostasis, thereby influencing morphological changes in cell colonies and biofilm detachment [9]. Similarly, prodigiosin secreted by *Serratia marcescens* acts as a paracrine signal within the colony, participating in the regulation of swarming motility and the remodeling of colony morphology [37,38].

Whether they are bacteriocins, iron carriers, or pigments, these secondary metabolites have demonstrated functions similar to those of signaling molecules in various ecological contexts. They blur the traditional boundaries between primary and secondary metabolism and between defense mechanisms and communication, demonstrating the high complexity and integration of microbial communication in nature.

## 3. Cross-Talk and Cascading Regulation in Integrated Signaling Networks

### 3.1. Hierarchical Regulation of Secondary Metabolism by the Classic QS System

In complex microbial communities, because the synthesis of secondary metabolites often involves significant metabolic energy expenditure, bacteria have evolved sophisticated multi-level regulatory networks. These networks ensure that bacteria synthesize these molecules in large quantities only when population density reaches a threshold, thereby maximizing ecological benefits [39]. Many gram-negative bacteria utilize multiple QS systems to achieve this control; these systems may have distinct or partially overlapping functions, and bacteria can combine them in an additive manner. Bacteria can also organize these systems into a hierarchical model, in which one system induces another [40].

**Figure 2:**
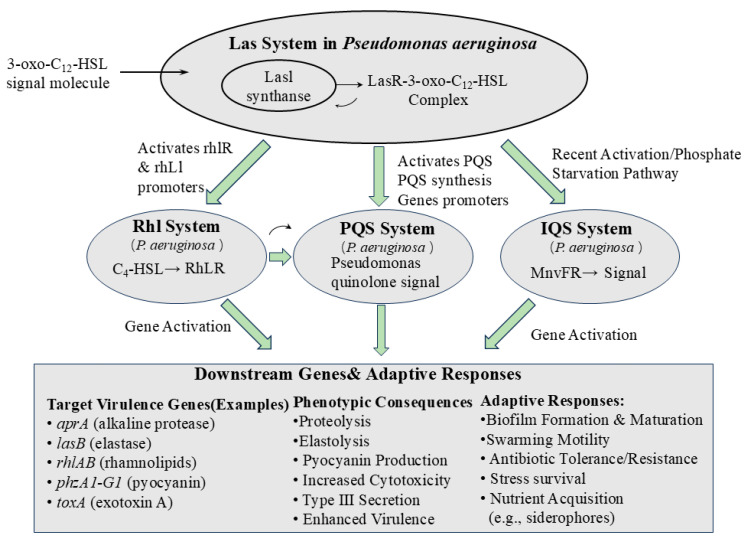
The hierarchical cascade regulatory network of *P. aeruginosa*. “Activate the rhlR & rhL1 promoters” is the caption for the green arrow.

Some bacteria utilize different systems to synthesize and recognize the same signaling molecule. *Erwinia carotovora* employs both the CarI/CarR and ExpI/ExpR systems, which are used to synthesize and recognize the OHHL (OC6-HSL) signal [40]. In another example, *Burkholderia plantarii* also possesses two distinct QS systems. One is an AHL-mediated system, in which AHL is synthesized by PlaI and detected by PlaR. The other is a DSF-mediated system [41], which uses RpfF as the synthase and RpfC, RpfG, or RpfR as receptors. Some bacteria, however, communicate by integrating multiple signals; the hierarchical cascade regulatory network of *P. aeruginosa* is the most classic example (Figure 2). In this network, the classical QS system at the top level acts as a master switch that initiates the synthesis of secondary metabolites. *P. aeruginosa* possesses four independent yet interdependent QS systems: the Las, Rhl, PQS, and the recently identified IQS systems. At the apex of this hierarchical network is the Las system, which initiates and regulates the signal transduction processes of the entire QS network [42]. As cell density increases, the signal molecule N-(3-oxododecanoyl)-L-hyaluronan (3-oxo-C12-HSL), synthesized by the signal synthetase LasI, accumulates extracellularly. When the concentration reaches a threshold, 3-oxo-C12-HSL returns to the cytoplasm and binds to its homologous receptor protein LasR, forming an activated transcriptional activation complex [43,44]. The LasR complex not only activates target virulence genes but also initiates downstream regulatory systems. It directly binds to the promoter regions of rhlR, rhlI, and PQS synthesis-related genes, thereby comprehensively activating the Rhl and PQS systems at the transcriptional level [42]. This hierarchical activation mechanism ensures bacterial adaptability under varying environmental conditions by progressively amplifying the initial weak environmental signal [44].

Through stepwise amplification by the Las system, the Rhl systems take over direct control of the biosynthesis of specific secondary metabolites. Rhamnolipids are potent biosurfactants that are essential for swarming motility and the formation of three-dimensional biofilm structures. The synthesis of rhamnolipids is directly regulated by the Rhl system; upon binding of RhlR to C4-HSL, this complex specifically binds to the rhlAB operon, directly initiating the expression of the major rhamnolipid synthase [45,46]. In contrast, the synthesis of the classic secondary metabolite pyocyanin involves cross-talk among multiple systems. The Las system indirectly controls pyocyanin production by activating the Rhl and PQS systems. PQS signaling molecules can specifically induce the *phz* operon, which controls the synthesis of phenazine compounds, while the RhlR-C4-HSL complex not only directly activates *phz* but also positively regulates the biological activity of PQS, forming a precise positive-feedback regulatory loop [42,47,48]. Global transcriptomic studies further confirm that the Las system, as a master regulator, indirectly or directly controls hundreds of genes involved in secondary metabolism and environmental adaptation through a cascade network [49]. Classic QS systems initiate the synthesis of secondary metabolites through a strict hierarchical cascade mechanism. This hierarchical regulation, comprising master switches, effector modules, and effector molecules, endows bacterial populations with exceptional decision-making stability and metabolic efficiency in complex and variable environments [42].

Of course, we must also recognize that the emergence of such a sophisticated network topology is no accident. Under the pressures of natural selection, the unrestrained synthesis of costly secondary metabolites would impose a significant fitness cost on the individual. Therefore, hierarchical regulation at the molecular level is essentially a survival strategy that has evolved to optimize resource allocation, ensuring that microorganisms strike a delicate balance between individual metabolic burden and competitive advantage within the population.

### 3.2. Feedback and Feedforward Control Networks in Collective Behavior

**Figure 3:**
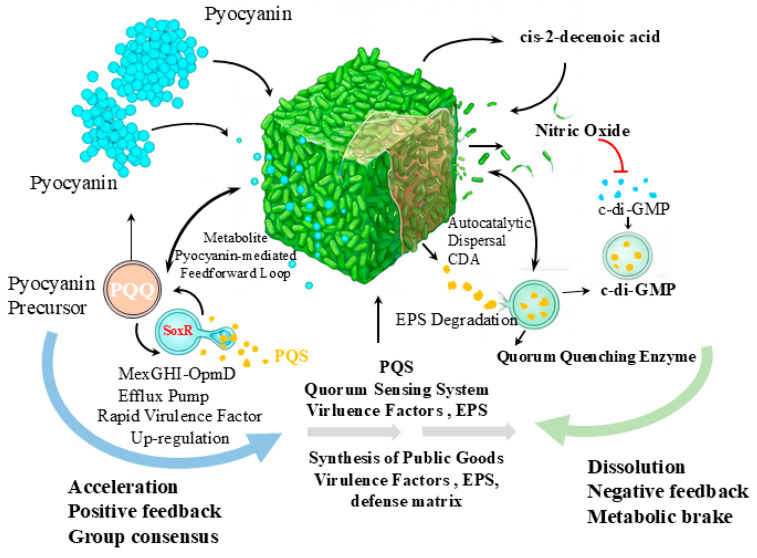
Integration of feedback and feedforward loops by secondary metabolites in microbial networks (e.g., *P. aeruginosa*). This figure shows two main antagonistic regulatory modules. Positive feedback module: Pyocyanin, its precursors, and the PQS quorum-sensing system are key factors. Result: Pyocyanin forms a positive feedback loop by activating the SoxR and MexGHI-OpmD efflux pumps, thereby accelerating the release of PQS signals. This drives the rapid upregulation of public goods such as virulence factors, defense matrix, and EPS, facilitating rapid consensus formation within the population and accelerating biofilm formation. Negative feedback module: CDA, NO and QQ enzymes are key factors. Result: To prevent excessive metabolic consumption, NO alters the cellular state by lowering c-di-GMP levels, CDA induces the degradation of EPS, and quorum-sensing enzymes attenuate the response signal. Together, these factors regulate metabolic homeostasis, triggering autocatalytic dispersion, which ultimately leads to the lysis of the biofilm and the dispersal of bacteria. The red lines indicate suppression or reduction. Blue arrows indicate positive feedback, green arrows indicate negative feedback, the gray arrows and modified black arrows have no specific meaning.

In the integrated signaling networks of microorganisms, unidirectional hierarchical regulation (Section 3.1) can only account for downstream signal transmission. However, to adapt to complex and dynamic environments and maintain metabolic equilibrium within the population, bacteria have evolved sophisticated feedback and feedforward loops (Figure 3). In these circuits, secondary metabolites at the network’s terminal nodes are no longer merely passive effectors; instead, they can exert a reverse influence on upstream QS systems, forming dynamic feedback loops [50]. This mechanism profoundly influences the acceleration, stabilization, or timely dissolution of collective behavior.

Positive feedback mechanisms are key to enabling microbial populations to rapidly reach a behavioral consensus when faced with environmental challenges. Studies have shown that after *P. aeruginosa* accumulates extracellularly and re-enters the cell, it can significantly alter the intracellular redox balance, directly activating the oxidative stress response transcription factor SoxR [51,52]. Activated SoxR upregulates the expression of the MexGHI-OpmD efflux pump, which serves as a critical transport pathway for PQS and its precursors [53]. This positive feedback loop greatly accelerates the activation of the PQS system. It initiates by the metabolite pyocyanin, mediated by the transcription factor SoxR and the efflux pump, and culminates in the massive secretion of the upstream QS signal PQS [54]. This mechanism ensures that, during critical stages of biofilm development or under competitive pressure, the entire population can rapidly and synchronously upregulate the synthesis of virulence factors and the defense matrix.

However, unchecked positive feedback activation leads to overproduction of public goods, and this high metabolic cost undoubtedly severely undermines the evolutionary fitness of individual organisms. To prevent excessive investment, microorganisms must possess a negative feedback mechanism that allows them to cease production at the appropriate time. The accumulation of certain secondary metabolites inhibits their own synthetic pathways; more importantly, specific metabolites act as negative feedback signals that directly trigger colony disaggregation. As biofilm density increases and internal nutrients are depleted, *P. aeruginosa* synthesizes a fatty acid-derived secondary metabolite—cis-2-decenoic acid (CDA). CDA not only alleviates metabolic stress caused by overcrowding but also acts as an endogenous dispersal signal to drive the degradation of extracellular polymeric substances (EPS), prompting cells to return to a free-swimming state [55]. Similarly, the endogenously produced small-molecule metabolite nitric oxide (NO) can, even at extremely low concentrations, disrupt the positive feedback loop sustaining biofilm formation by enhancing the activity of intracellular cyclic diguanylate (c-di-GMP) hydrolases, thereby acting as a negative feedback mechanism to induce biofilm disaggregation [56]. Furthermore, during the late stages of QS activation, some bacteria endogenously upregulate QQ enzyme expression to degrade QS signaling molecules actively, thereby preventing resource wastage caused by excessive signaling [57,58].

Secondary metabolites are deeply integrated into QS networks through intricate positive, negative, and feedforward loops. Positive feedback accelerates the establishment of collective coordination, while negative feedback acts as a safety valve, preventing metabolic collapse within the population and granting it the flexibility to seek new habitats [59]. The existence of these loops confirms that the complex topological structure of molecular networks is precisely the mechanism evolved to optimize resource allocation under the pressures of ruthless natural selection.

### 3.3. Secondary Metabolite-Driven Interspecies Communication and Information Interference

In nature, microorganisms rarely exist as single, pure cultures; instead, they inhabit complex microecosystems where multiple species coexist. In such densely populated environments, secondary metabolites are no longer merely intraspecies communications between cells of the same species, but rather a universal language that transcends species boundaries. Through interspecies cross-talk and the recognition of chemical signals, microorganisms can not only detect the presence of competitors or partners but also use this information as a tool for competition, dynamically adjusting their ecological strategies [60,61].

Secondary metabolites produced by different microorganisms can often directly interfere with or reprogram the QS networks of other species. In co-infection models involving the human respiratory tract or wounds, 4-hydroxy-2-heptylquinoline N-oxide derived from *P. aeruginosa* not only serves as a precursor for its own secondary metabolism but also effectively inhibits the cytochrome-based respiratory chain of *Staphylococcus aureus*. This sublethal attack acts as a stress signal recognized by *Staphylococcus aureus*, leading to altered expression patterns of its virulence factors and promoting a shift from a rapidly growing state to a persistent, hard-to-eradicate small-colony variant [62,63]. Similarly, certain ubiquitous commensal bacteria, such as those of the genus *Bacillus*, can secrete QQ enzymes as secondary metabolites [64]. These molecules degrade the AHL signaling relied upon by pathogenic bacteria, rendering their QS networks unresponsive, thereby successfully attenuating their virulence and initiating a competition for survival [65].

In community information games, recognizing competitors’ secondary metabolite signals is key to gaining a survival advantage. By detecting autoinducers released by other species, microorganisms can anticipate competitive pressure or resource availability in advance, thereby preparing for defense or counterattack [66]. The opportunistic pathogen *Burkholderia cepacia* frequently coexists with *P. aeruginosa* in the lungs of patients with cystic fibrosis. Although *Burkholderia cepacia* possesses its own QS system, its receptor protein CepR responds with high sensitivity to AHL signals secreted by *P. aeruginosa*. Upon receiving the signal, *Burkholderia cepacia* interprets it as an indicator of a high-density environment, preemptively activating the synthesis of virulence factors and competitive secondary metabolites to gain a competitive advantage in the habitat [67,68]. Close chemical interactions also exist between fungi and bacteria. Farnesol, an alcohol-based secondary metabolite secreted by *Candida albicans*, not only regulates its own hyphal-to-yeast phase transition but is also recognized by *P. aeruginosa*. Upon recognizing farnesol, *P. aeruginosa* significantly downregulates pyocyanin synthesis, thereby optimizing energy expenditure and enhancing its competitive mechanisms against fungi [69,70]. Such mechanisms demonstrate that, through long-term evolutionary conflict, microorganisms have constructed complex networks of cooperation and competition based on the multifunctionality of signaling molecules, exhibiting remarkable ecological adaptability [71].

### 3.4. The Concentration-Dependent Signaling Hub Framework for Secondary Metabolites

Synthesizing the evidence presented across Section 2 and Section 3, a unifying principle emerges: secondary metabolites function as concentration-dependent signaling hubs that integrate environmental information and orchestrate collective bacterial behavior across multiple scales. This concept is characterized by the following key conclusions, summarized in Table 1.

These six characteristics collectively establish secondary metabolites not as downstream effectors of linear signaling pathways but as central nodes within integrated, multilayered networks. This framework extends beyond the *P. aeruginosa*-centric model that has historically dominated QS research, encompassing diverse Gram-positive systems and interspecies interactions that bridge bacterial phyla. The concentration-dependent signaling hub concept provides a coherent theoretical lens through which to interpret the dual-function phenomena described throughout this review and offers a foundation for designing network-based interventions in antimicrobial therapy, environmental bioremediation, and industrial fermentation.

## 4. The Ecology and Evolutionary Dynamics of Dual-Function Metabolic Molecules

### 4.1. Synergistic Antibacterial Mechanisms at Critical Density

As mentioned earlier, secondary metabolites such as broad-spectrum antibiotics, toxins, and tissue-degrading enzymes are powerful biochemical weapons. However, the synthesis and secretion of these large molecules or complex secondary metabolites inevitably impose significant adaptive costs on individual cells [72]. If bacteria secrete such substances at low population densities, not only is their efficacy low, but they are also highly susceptible to elimination by natural selection due to metabolic disadvantages [73,74]. As discussed in Section 3, to circumvent this metabolic disadvantage, natural selection has driven the evolution of the QS system as a coordination hub that precisely evaluates the return on investment. The QS network enables individual cells to quantify population density and mass-transfer constraints within the microenvironment continuously; only when the concentration of self-activating signaling molecules exceeds a critical threshold does the entire population reach consensus and synchronously initiate gene expression for secondary metabolite synthesis [75]. Cornforth et al. found that when *P. aeruginosa* infects a host, it first accumulates to sufficient numbers through silent proliferation; once the density threshold is exceeded, the population instantaneously and synchronously releases large amounts of pyocyanin, elastase, and rhamnolipids via the QS system [76]. This sudden, high-concentration release of virulence factors ensures that effector molecules rapidly establish a concentration advantage in the local microenvironment, thereby effectively breaching the host’s defensive barriers [3].

This ecological strategy was later conceptualized as “efficiency sensing”. According to this theory, bacteria invest in signaling pathways to ensure that the QS molecules they secrete are effectively retained in the microenvironment, thereby optimizing the fitness gains derived from metabolic expenditure [77,78]. Both theoretical models and experiments confirm that, in pathogen-host interactions, the dynamic balance between population density and virulence determines the pathogen’s survival. By using QS systems to delay and synchronize the secretion of signaling molecules, bacteria successfully optimize energy allocation, ensuring that metabolic investments translate into a major competitive advantage for the population within complex ecological niches [74,79,80]. High-cost signaling is essentially designed to avoid unnecessary expenditure. Through QS-mediated coordination mechanisms, microorganisms ingeniously aggregate the extremely limited biochemical capabilities of individuals into a powerful force that erupts at a critical density, thereby maximizing the return on investment in public goods within the harsh context of natural evolution.

### 4.2. Public Goods Management and Mechanisms for Collective Maintenance

In microbial communities, the secretion of secondary metabolites via QS is a classic example of public good provision; however, this costly metabolic investment inevitably faces a problem: the emergence of non-cooperative individuals with genetic mutations within the population. These individuals do not expend their own energy to produce public goods but can still reap the benefits of resources secreted by cooperators, thereby gaining a growth advantage and potentially leading to a decline in the fitness of the entire population [81,82,83]. To counter this selective pressure, microorganisms have evolved metabolic regulatory mechanisms based on pleiotropic constraints [84,85,86].

Many secondary metabolites and signaling molecules that form the core of the QS network are not merely carriers of information but also possess potent intrinsic toxicity. It is precisely this dual function that serves as a barrier to stable cooperative behavior. In *P. aeruginosa*, the PQS serves not only as a QS molecule that drives the expression of various virulence factors but also as a potent pro-oxidant capable of inducing significant oxidative stress toxicity in the bacteria themselves [87]. When wild-type cooperative strains produce and respond to PQS, they not only activate the release of public goods but also trigger antioxidant defense mechanisms downstream in the molecular pathway. In contrast, non-cooperative mutant strains that have lost their QS receptors or signaling responsiveness cannot activate this antioxidant feedback loop; consequently, when exposed to PQS produced by wild-type strains, they suffer severe oxidative damage and growth inhibition [88]. In addition to the toxicity of the signaling molecules themselves, toxic secondary metabolites regulated by these signaling molecules also play a role in metabolic regulation. Research by Wang et al. indicates that the public products of *P. aeruginosa*, which are strictly regulated by QS, include highly toxic hydrogen cyanide (HCN). In mixed-culture microecosystems, while the wild-type population secretes HCN, it also expresses specific anti-cyanide respiratory chain enzymes to protect itself; whereas individuals attempting to conserve energy by suppressing the QS system lose their resistance to HCN and are subject to specific chemical elimination within the population [89].

Furthermore, the evolutionary mechanism that combines cooperative signaling with lethal secondary metabolites is not limited to Gram-negative bacteria; it is particularly evident in the phenomenon of intraspecific competition in the Gram-positive bacterium *Streptococcus pneumoniae*. In this species, high cell density triggers the accumulation of cytotoxic signaling peptides (CSPs). When CSP reaches a critical threshold, it not only induces genetic transformability but also activates the secretion of lysozyme and bacteriocins. Cooperative cells that actively respond to the CSP signal collectively express immune proteins to protect themselves, while non-responsive variants or non-cooperative individuals within the population are lysed by these secondary metabolites. This targeted cell lysis releases DNA and nutrients, thereby benefiting the cooperative survivors. This cross-phylum mechanism confirms that the use of toxic metabolites to constrain non-cooperators and maintain population stability is a widely favored evolutionary network logic under natural selection [90,91].

**Figure 4:**
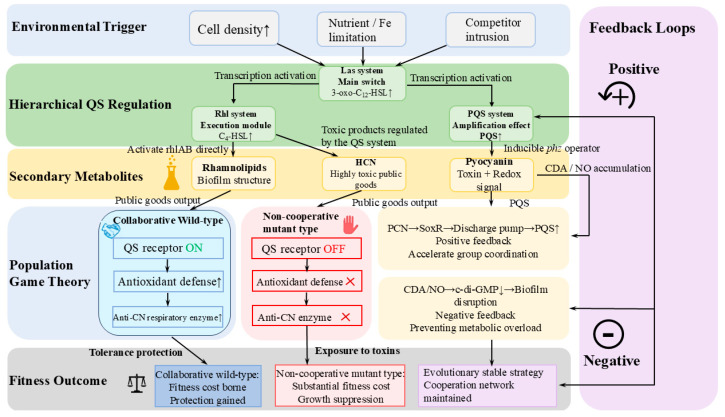
A conceptual model based on *P. aeruginosa* that links molecular regulatory networks to the coevolution of microorganisms. (Solid arrow: activation/upregulation, ↑: increase, ↓: decrease, ×: missing features, PCN: pyocyanin, CDA: cis-2-decenoic acid, NO: nitric oxide).

To systematically elucidate this evolutionary strategy, Figure 4 presents a conceptual model that links these underlying molecular mechanisms to evolutionary stability at the population level. As shown in the figure, the tight coupling between public good secretion and self-tolerance pathways creates an exclusive fitness advantage for wild-type cooperators. This evolutionary strategy, which rigidly couples cooperative signals with genes for self-tolerance through a single regulatory hub, establishes strong selective pressure against non-cooperative individuals, thereby ensuring the long-term evolutionary stability of the signaling network [92].

### 4.3. The Process of Pre-Adaptation and Coevolution of Bifunctional Molecules

As mentioned earlier, a key evolutionary reason why microorganisms invest in synthesizing secondary metabolites or signaling molecules is that these molecules often possess dual functions and pleiotropy. This trait not only provides microorganisms with great flexibility for survival in complex environments but also, through preadaptation mechanisms, constitutes an important driving force for coevolution among species [85,86,93]. Evolutionary biology suggests that a trait with a specific physiological function may acquire entirely new functions when the environment changes. In microbial signaling networks, many signaling molecules did not initially evolve for communication but rather to address fundamental physiological and ecological challenges [94]. From a physiological perspective, pyocyanin is a highly efficient electron shuttle and antibiotic; it helps bacteria maintain intracellular redox balance in anaerobic environments while suppressing spatial competitors [9]. Over the course of long-term evolution, this secondary metabolite with strong redox activity has been pre-adapted as a QS signaling molecule that specifically binds transcription factors, thereby regulating population behavior. This dual function of secondary metabolites confers great ecological flexibility on bacteria: under metabolic stress, they serve as survival tools, while during population expansion, they act as coordination signals. Rather than evolving entirely new, single-function molecules specifically for communication, bacteria repurpose existing metabolic substances, thereby significantly improving energy efficiency [95].

The dual functions of secondary metabolites not only play a role within a single species but also serve as catalysts for coevolution across multi-species communities. In natural environments, antibiotics secreted by a microorganism often transform into interspecies signaling molecules that regulate gene expression when they diffuse to distant locations and reach sublethal concentrations [96,97]. When Species A releases molecules that serve both toxic and signaling functions, Species B, occupying the same ecological niche, evolves corresponding signal-recognition and detoxification mechanisms in response to this selective pressure. By responding to low-concentration secondary metabolite signals from Species A and integrating them into its own regulatory network, Species B preemptively activates efflux pumps, membrane formation, or the production of degradative enzymes to make adaptive adjustments [98,99]. This interaction forms a dynamic feedback loop: A’s substances drive B to evolve more sensitive sensing and defense mechanisms; B’s resistance, in turn, compels A to modify the structure of its secondary metabolites further to maintain toxicity or signaling specificity [100]. In this process, signaling molecules not only maintain intraspecific order but also shape highly intricate, mutually balancing microecological networks at the community level.

Microbial metabolic investment in signaling networks is not an evolutionary redundancy; rather, it is an ecological strategy grounded in fitness. The dual functions and preadaptation of molecules not only endow individual cells with metabolic flexibility in dynamic environments but also provide the chemical foundation for the coevolution of complex communities. It is precisely through this mechanism, which integrates functional molecules with regulatory networks, that microorganisms have established sophisticated ecological adaptation networks under natural selection [76].

## 5. Intervention Strategies and Applications Targeting Signal Transduction Networks

### 5.1. Novel Ecological Intervention Strategies Based on Signal Network Remodeling

In the face of the increasingly severe crisis of AMR, QQ targeting microbial communication systems has been viewed as highly promising. Traditional QQ primarily disrupts pathogen communication networks by enzymatically degrading signaling molecules or by using receptor antagonists, thereby inhibiting the secretion of virulence factors [65,101]. This approach can significantly reduce pathogenicity without directly killing bacteria and alleviate selection pressure theoretically. But the latest evolutionary models suggest that simple communication blockade may still be circumvented by microorganisms through receptor mutations or signal network reorganization [102]. Therefore, based on a deep understanding of microbial ecology and the coordination mechanisms and public goods management discussed above, a new generation of anti-virulence strategies is evolving from passive communication disruption toward active remodeling of pathogen signaling networks.

Recalling the coordination mechanisms described in Section 4.1, microorganisms rely on QS networks to accurately assess whether critical density has been reached before synchronously secreting costly secondary metabolites and virulence factors. Disrupting this precise regulatory mechanism is key to novel interventions: by artificially introducing large amounts of exogenous activators or highly affine signal mimetics into the infection microenvironment, one can artificially generate a signal indicating high population density [92]. When pathogens receive this spurious signal at extremely low densities or during the early stages of infection, they incur a high fitness cost. At this point, cells maladaptively over-activate transcriptional cascades, leading to the synthesis of costly secondary metabolites [103]. Since the actual population has not reached the critical density, these prematurely secreted public goods are rapidly diluted in the host’s body fluids, losing their original efficacy, and also trigger a severe decline in fitness. The immense metabolic burden rapidly depletes the individual cell’s energy reserves, severely limiting its division and proliferation rates. Furthermore, these prematurely exposed molecules trigger alertness and clearance responses from the host immune system. This ecological intervention strategy, which exploits the pathogen’s social coordination mechanisms, effectively weakens the pathogen’s survival fitness in the host microenvironment by drastically increasing its metabolic load [104].

In addition to metabolic exhaustion, another approach is the dual function of signaling networks described in Section 4.2 and Section 4.3. Since many signaling molecules possess strong intrinsic toxicity, Mühlen and Dersch found that overstimulating the synthetic pathways of these molecules through synthetic biology or specific small-molecule activators can convert them into biochemical inhibitory pathways targeting the pathogens themselves [105]. This approach directly exploits the bacterial metabolic regulatory system, causing normal cells to suffer severe oxidative stress when they overproduce toxic signaling molecules. Evolutionary medicine has also proposed an intervention strategy based on ecological selection: artificially introducing or supporting genetically engineered non-responsive individuals into clinical infection foci. These individuals lack a response to the targeted signals but can gain a niche advantage by exploiting resources. This parasitic and competitive behavior disrupts the original metabolic balance and may ultimately trigger a systemic decline in the pathogen population’s fitness [106,107].

Importantly, ecological intervention strategies must be tailored to the specific topological logic of the target pathogen’s signaling network. While inducing premature activation is effective against pathogens that exhibit virulence at high densities, the opposite strategy—using quorum-sensing agonists—is highly effective against pathogens with negatively regulated networks. In *Vibrio cholerae*, high-density quorum-sensing signaling actively suppresses the expression of virulence factors and drives biofilm disaggregation to facilitate dissemination. Therefore, the artificial administration of QS receptor agonists can trick *Vibrio cholerae* into perceiving high cell density early in the infection. This artificially forces the pathogen to prematurely shut down its pathogenicity cascade and detach from the host intestine, thereby clearing the infection without the survival pressure typically associated with traditional antibiotics [108,109]. This highlights the immense potential of network-specific modulation compared to broad-spectrum signal blockade.

### 5.2. Antiviral Intervention Strategies Targeting Network Decoupling

As mentioned earlier, QS networks not only regulate public goods but also exhibit highly complex pleiotropy, tightly coupling the secretion of bacterial virulence factors with biofilm formation. Traditional QQ attempts to silence pathogens by completely blocking upstream signaling; however, this approach tends to exert strong selective pressure on the entire bacterial community network, which may subsequently drive the emergence of drug resistance or network reorganization [110]. Therefore, new anti-virulence strategies advocate for a systems biology approach, designing small molecules that can specifically intervene at key downstream nodes within the signaling network to achieve precise decoupling of pathogenic phenotypes [111].

In many pathogenic bacteria, c-di-GMP acts as a key second messenger, serving as a molecular switch between virulence expression and biofilm formation. During natural evolution, QS signals typically intersect with the c-di-GMP metabolic pathway, ensuring that bacteria moderately downregulate certain acute virulence factors when c-di-GMP levels are high, thereby establishing chronic infections [112,113]. New anti-virulence strategies utilize small-molecule compounds, such as specific synthase inhibitors, to target this critical juncture precisely. By artificially reducing intracellular c-di-GMP levels, these agents can induce bacteria to disaggregate from biofilm structures and revert to a free-floating state [114]. More importantly, this intervention does not directly activate upstream acute virulence signaling pathways, thereby successfully decoupling biofilm disassembly from virulence outbreaks—two phenotypes that were previously coupled. Pathogens that have lost their physical barrier and have not activated virulence factor expression are highly susceptible to elimination by the host’s immune system or conventional antibiotics [115]. Another decoupling strategy involves directly targeting downstream transcription factors that regulate specific virulence factors, without interfering with the primary signaling network’s basal metabolic functions. Studies have demonstrated that small molecules such as savirin can specifically bind to the AgrA regulator in *Staphylococcus aureus*, blocking the upregulation of tissue-degrading enzymes without significantly disrupting bacterial proliferation or basal metabolic networks [116,117].

Regarding the PQS system of *P. aeruginosa*, recent studies have designed small-molecule antagonists that specifically target the PqsR receptor. Because PQS molecules possess dual properties of signal transduction and intrinsic toxicity, the specific inhibition of PqsR not only reduces the production of virulence factors such as pyocyanin but also decouples the bacterial population’s tolerance to strong oxidative stress [118]. This decoupling strategy aims to circumvent the intense natural selection triggered by traditional antibiotics and undermines the multifaceted protective mechanisms pathogens rely on to evade immune clearance.

Although network rewiring and decoupling interventions theoretically significantly reduce direct selective pressures on cell survival, this does not mean they are completely immune to the evolution of drug resistance. In the long-term evolutionary game, pathogens exposed to sustained small-molecule inhibitors may still reduce drug affinity through receptor gene mutations. Therefore, before promoting novel ecological intervention therapies, it is necessary to establish systematic models for monitoring coevolutionary dynamics to fully assess the long-term evolutionary risks they may induce in complex clinical environments.

### 5.3. Industrial Applications of QS Networks in the Dynamic Regulation of Fermentation

In the previous section, we discussed how microorganisms use QS networks to precisely assess population density, thereby initiating the synthesis of secondary metabolites at the optimal time while avoiding excessive metabolic burden. This evolutionarily developed mechanism for decoupling growth from production provides an extremely valuable blueprint for modern industrial biotechnology and metabolic engineering [119].

In natural QS circuits, the accumulation rate of AHL-type signaling molecules directly determines the timing of activation of genes encoding secondary metabolites. Research by Tsao et al. demonstrated that by modulating the expression levels of AHL synthase genes, the induction threshold can be artificially set, thereby precisely controlling the time window for the initiation of metabolite production, such as antibiotics [120]. In recent years, Dinh et al. further utilized synthetic biology tools to design programmable QS oscillatory circuits, achieving periodic pulsed production of secondary metabolites in *E. coli*, effectively circumventing the metabolic stress associated with continuous high-level expression [121]. In natural producers of complex secondary metabolites, such as *Streptomyces*, the endogenous signaling networks are inherently extremely complex. The latest industrial approach involves using gene-editing tools such as CRISPR to precisely reconfigure the cascade amplification effects of these endogenous QS nodes, thereby lifting feedback inhibition caused by intrinsic metabolic regulation and unlocking and overexpressing the biosynthetic gene clusters of natural antibiotics [122]. Furthermore, the industry is extending single-species signal regulation to artificial co-culture systems involving multiple microbial communities. Drawing on the mechanisms of interspecies communication and coevolution in microecosystems described in Section 4.3, researchers have established artificial cross-species communication between different engineered strains using orthogonal QS signaling systems. By decomposing complex metabolic pathways, which are often lengthy, cytotoxic, or highly coenzyme-consuming, and assigning them to different microorganisms for execution, an efficient division of labor can be achieved [123,124]. In this system, signaling molecules not only regulate the synthesis of individual compounds but also function as a central regulatory hub that coordinates the overall process. By adjusting the expression ratios of enzymes at each step based on real-time metabolic feedback from different microbial populations, this approach overcomes the metabolic bottlenecks inherent to a single host [125].

## 6. Conclusions

Reconfiguring secondary metabolites and QS networks into a dynamic, integrated framework represents an expansion of our understanding of microbial collective behavior. Classic linear communication models and the single-antibiotic hypothesis cannot fully explain the systemic resilience of pathogens when facing host clearance and competition within the microbiome. By establishing the concept of “Concentration-Dependent Signaling Hubs”, this review draws several core scientific conclusions:

A paradigm shift in chemical ecology: Secondary metabolites are not merely end products or isolated chemical defense factors; rather, their concentration-dependent dual functionality enables them to act as central nodes in metabolic networks, precisely balancing metabolic costs and ecological benefits under dynamic environmental stresses.

A universal cross-phylogenetic evolutionary logic: The use of secondary metabolites as regulatory hubs is not a species-specific trait unique to *P. aeruginosa*, but rather a highly conserved evolutionary strategy widely present across various phylogenetic lineages, spanning Gram-negative and Gram-positive bacteria, and even communities across different kingdoms.

New entry points for precise intervention: Examining chemical ecology through the lens of dynamic signaling networks can reveal new vulnerabilities. Future anti-pathogenic therapies need not attempt to broadly inhibit signal transduction but should instead focus on “network decoupling” and leverage the inherent toxicity of these hubs to address AMR.

Moving forward, quantifying local gradients of sub-inhibitory concentrations in complex habitats remains a challenge. However, breakthroughs in spatial single-cell transcriptomics and ultra-high-resolution mass spectrometry imaging will enable us to characterize these signaling hubs in situ. Ultimately, this unified systems-biology perspective not only advances fundamental evolutionary ecology but also establishes a critical theoretical foundation for microbiome modulation, environmental bioremediation, and autonomous industrial synthetic biology.

## Figures and Tables

**Table 1:** Key characteristics of secondary metabolites as concentration-dependent signaling hubs.

Core Principle	Mechanistic Basis	Representative Examples	Ecological Consequence
Bifunctionality	The same molecule acts as a signal at subinhibitory concentrations and as a weapon at high concentrations.	Pyocyanin (*P. aeruginosa*), surfactin (*B. subtilis*), γ-butyrolactones (*Streptomyces* spp.), nisin (*L. lactis*)	Maximizes ecological versatility while minimizing metabolic cost.
Threshold dependence	Signaling function is activated only above a critical concentration, often via bistable or switch-like regulatory circuits.	SCB1-ScbR bistable switch (*S. coelicolor*), Las/Rhl QS hierarchy (*P. aeruginosa*)	Acts as a noise filter; ensures collective commitment only under favorable conditions.
Hierarchical integration	Secondary metabolite synthesis is controlled by upstream QS systems, and metabolites in turn exert feedback on those systems.	PQS positive feedback via SoxR (*P. aeruginosa*), CDA/NO negative feedback triggering biofilm dispersal	Enables dynamic adaptation; prevents metabolic runaway.
Interspecies cross-talk	Secondary metabolites from one species modulate QS networks, biofilm formation, or stress responses of neighboring species.	HQNO from *P. aeruginosa* inducing *S. aureus* SCV formation; plant metabolites inhibiting *E. coli* biofilms via QQ	Shapes community structure and competitive hierarchies.
Evolutionary coupling	Dual-function molecules couple cooperative public-good secretion with private self-tolerance mechanisms.	PQS toxicity selecting against QS-defective cheaters (*P. aeruginosa*); HCN policing mechanism	Stabilizes cooperation and prevents social cheating.
Widespread phylogenetic distribution	Functional analogues of concentration-dependent signaling hubs exist across Gram-negative and Gram-positive bacteria, as well as in fungi.	GBL systems in *Streptomyces* spp., AIP-based QS in *S. aureus*, farnesol in *Candida albicans*	Indicates convergent evolution of this regulatory logic.

## Data Availability

No new data were created or analyzed in this study. Data sharing is not applicable to this article.
